# Supplementary material for: The Potential of Co-Evolution and Interactions of Gut Bacteria–Phages in Bamboo-Eating Pandas: Insights from Dietary Preference-Based Metagenomic Analysis
Source: Microorganisms. 2024 Mar 31;12(4):713. doi: 10.3390/microorganisms12040713 (PMC11051890; doi:10.3390/microorganisms12040713)
Supplement: Supplementary file 1 [file microorganisms-12-00713-s001.zip › microorganisms-2850991-supplementary.pdf]

Supporting information

Table S1 Information on the macrogenomic samples used in this study.

Sample ID	Group	Dietary preference	Publication time	Citation
CA_1	CA	Carnivores	2018	(Zhu et al., 2018a)
CA_2	CA	Carnivores	2018	(Zhu et al., 2018a)
CA_3	CA	Carnivores	2018	(Zhu et al., 2018a)
CA_4	CA	Carnivores	2018	(Zhu et al., 2018a)
CA_5	CA	Carnivores	2018	(Zhu et al., 2018a)
CA_6	CA	Carnivores	2018	(Zhu et al., 2018a)
CA_7	CA	Carnivores	2018	(Zhu et al., 2018a)
CA_8	CA	Carnivores	2018	(Zhu et al., 2018a)
CA_9	CA	Carnivores	2018	(Zhu et al., 2018a)
CA_10	CA	Carnivores	2018	(Zhu et al., 2018a)
CA_11	CA	Carnivores	2018	(Zhu et al., 2018a)
CA_12	CA	Carnivores	2018	(Zhu et al., 2018a)
CA_13	CA	Carnivores	2018	(Zhu et al., 2018a)
CA_14	CA	Carnivores	2018	(Zhu et al., 2018a)
CA_15	CA	Carnivores	2018	(Zhu et al., 2018a)
CA_16	CA	Carnivores	2018	(Zhu et al., 2018a)
CA_17	CA	Carnivores	2018	(Zhu et al., 2018a)
CA_18	CA	Carnivores	2018	(Zhu et al., 2018a)
CA_19	CA	Carnivores	2018	(Zhu et al., 2018a)
HE_1	HE	Herbivores	2018	(Zhu et al., 2018a)
HE_2	HE	Herbivores	2018	(Zhu et al., 2018a)
HE_3	HE	Herbivores	2018	(Zhu et al., 2018a)
HE_4	HE	Herbivores	2018	(Zhu et al., 2018a)
HE_5	HE	Herbivores	2018	(Zhu et al., 2018a)
HE_6	HE	Herbivores	2018	(Zhu et al., 2018a)
HE_7	HE	Herbivores	2018	(Zhu et al., 2018a)
HE_8	HE	Herbivores	2018	(Zhu et al., 2018a)
HE_9	HE	Herbivores	2018	(Zhu et al., 2018a)
HE_10	HE	Herbivores	2018	(Zhu et al., 2018a)
HE_11	HE	Herbivores	2018	(Zhu et al., 2018a)
HE_12	HE	Herbivores	2018	(Zhu et al., 2018a)
OC_1	OC	Omnivorous	2018	(Zhu et al., 2018a)
OC_2	OC	Omnivorous	2018	(Zhu et al., 2018a)
OC_3	OC	Omnivorous	2018	(Zhu et al., 2018a)
OC_5	OC	Omnivorous	2018	(Zhu et al., 2018a)
OC_6	OC	Omnivorous	2018	(Zhu et al., 2018a)
OC_7	OC	Omnivorous	2018	(Zhu et al., 2018a)

OC_8	OC	Omnivorous	2018	(Zhu et al., 2018a)
OC_9	OC	Omnivorous	2018	(Zhu et al., 2018a)
OC_10	OC	Omnivorous	2018	(Zhu et al., 2018a)
OC_11	OC	Omnivorous	2018	(Zhu et al., 2018a)
Chengdu_1	GPCD	Bamboo-eating giant pandas	2018	(Zhang et al., 2018)
Chengdu_2	GPCD	Bamboo-eating giant pandas	2018	(Zhang et al., 2018)
Chengdu_3	GPCD	Bamboo-eating giant pandas	2018	(Zhang et al., 2018)
Chengdu_4	GPCD	Bamboo-eating giant pandas	2018	(Zhang et al., 2018)
Chengdu_5	GPCD	Bamboo-eating giant pandas	2018	(Zhang et al., 2018)
Chengdu_6	GPCD	Bamboo-eating giant pandas	2018	(Zhang et al., 2018)
Chengdu_7	GPCD	Bamboo-eating giant pandas	2018	(Zhang et al., 2018)
Yaan_1	GPYA	Bamboo-eating giant pandas	2019	(Guo et al., 2019)
Yaan_2	GPYA	Bamboo-eating giant pandas	2019	(Guo et al., 2019)
Yaan_3	GPYA	Bamboo-eating giant pandas	2019	(Guo et al., 2019)
Yaan_4	GPYA	Bamboo-eating giant pandas	2019	(Guo et al., 2019)
Yaan_5	GPYA	Bamboo-eating giant pandas	2019	(Guo et al., 2019)
Yaan_6	GPYA	Bamboo-eating giant pandas	2019	(Guo et al., 2019)
Yaan_7	GPYA	Bamboo-eating giant pandas	2019	(Guo et al., 2019)
Yaan_8	GPYA	Bamboo-eating giant pandas	2019	(Guo et al., 2019)
Yaan_9	GPYA	Bamboo-eating giant pandas	2019	(Guo et al., 2019)
Yaan_10	GPYA	Bamboo-eating giant pandas	2019	(Guo et al., 2019)
Qinling_1	GPQIN	Bamboo-eating giant pandas	2017	(Wu et al., 2017)
Qinling_3	GPQIN	Bamboo-eating giant pandas	2017	(Wu et al., 2017)
Qinling_4	GPQIN	Bamboo-eating giant pandas	2017	(Wu et al., 2017)
Qinling_5	GPQIN	Bamboo-eating giant pandas	2017	(Wu et al., 2017)
Qinling_6	GPQIN	Bamboo-eating giant pandas	2017	(Wu et al., 2017)
Qinling_7	GPQIN	Bamboo-eating giant pandas	2017	(Wu et al., 2017)
Qinling_8	GPQIN	Bamboo-eating giant pandas	2017	(Wu et al., 2017)
Qinling_9	GPQIN	Bamboo-eating giant pandas	2017	(Wu et al., 2017)
Qinling_10	GPQIN	Bamboo-eating giant pandas	2017	(Wu et al., 2017)
Qionglai_1	GPQIO	Bamboo-eating giant pandas	2019	(Guo et al., 2019)
Qionglai_2	GPQIO	Bamboo-eating giant pandas	2019	(Guo et al., 2019)
Qionglai_3	GPQIO	Bamboo-eating giant pandas	2019	(Guo et al., 2019)
Qionglai_4	GPQIO	Bamboo-eating giant pandas	2019	(Guo et al., 2019)
Qionglai_5	GPQIO	Bamboo-eating giant pandas	2019	(Guo et al., 2019)
Qionglai_6	GPQIO	Bamboo-eating giant pandas	2019	(Guo et al., 2019)
Qionglai_7	GPQIO	Bamboo-eating giant pandas	2019	(Guo et al., 2019)
XXL_1	GPXXL	Bamboo-eating giant pandas	2018	(Zhu et al., 2018b)
XXL_2	GPXXL	Bamboo-eating giant pandas	2018	(Zhu et al., 2018b)
XXL_3	GPXXL	Bamboo-eating giant pandas	2018	(Zhu et al., 2018b)
XXL_4	GPXXL	Bamboo-eating giant pandas	2018	(Zhu et al., 2018b)
XXL_5	GPXXL	Bamboo-eating giant pandas	2018	(Zhu et al., 2018b)
XXL_6	GPXXL	Bamboo-eating giant pandas	2018	(Zhu et al., 2018b)
XXL_7	GPXXL	Bamboo-eating giant pandas	2018	(Zhu et al., 2018b)

XXL_8	GPXXL	Bamboo-eating giant pandas	2018	(Zhu et al., 2018b)
XXL_9	GPXXL	Bamboo-eating giant pandas	2018	(Zhu et al., 2018b)
XXL_10	GPXXL	Bamboo-eating giant pandas	2018	(Zhu et al., 2018b)
XXL_11	GPXXL	Bamboo-eating giant pandas	2018	(Zhu et al., 2018b)
XXL_12	GPXXL	Bamboo-eating giant pandas	2018	(Zhu et al., 2018b)
XXL_13	GPXXL	Bamboo-eating giant pandas	2018	(Zhu et al., 2018b)
XXL_14	GPXXL	Bamboo-eating giant pandas	2018	(Zhu et al., 2018b)
XXL_15	GPXXL	Bamboo-eating giant pandas	2018	(Zhu et al., 2018b)
XXL_16	GPXXL	Bamboo-eating giant pandas	2018	(Zhu et al., 2018b)
XXL_17	GPXXL	Bamboo-eating giant pandas	2018	(Zhu et al., 2018b)
XXL_18	GPXXL	Bamboo-eating giant pandas	2018	(Zhu et al., 2018b)
XXL_19	GPXXL	Bamboo-eating giant pandas	2018	(Zhu et al., 2018b)
RPxxl_1	RP	Bamboo-eating red pandas	2018	(Zhu et al., 2018b)
RPxxl_2	RP	Bamboo-eating red pandas	2018	(Zhu et al., 2018b)
RPxxl_3	RP	Bamboo-eating red pandas	2018	(Zhu et al., 2018b)
RPxxl_4	RP	Bamboo-eating red pandas	2018	(Zhu et al., 2018b)
RPxxl_5	RP	Bamboo-eating red pandas	2018	(Zhu et al., 2018b)
RPxxl_6	RP	Bamboo-eating red pandas	2018	(Zhu et al., 2018b)

Table S2 Information on the species and their groupings used in this study.

Species	Scientific name	Order	Family	Group
Giant panda	*Ailuropoda melanoleuca*	Carnivora	Ursidae	GP
Red panda	*Ailurus fulgens*	Carnivora	Ailuridae	RP
Maned wolf	*Chrysocyon brachyurus*	Carnivora	Canidae	CA
Arctic fox	*Vulpes lagopus*	Carnivora	Canidae	CA
Silver fox	*Vulpes vulpes*	Carnivora	Canidae	CA
Corsac fox	*Vulpes corsac*	Carnivora	Canidae	CA
Red fox	*Vulpes vulpes*	Carnivora	Canidae	CA
Dhole	*Cuon alpinus*	Carnivora	Canidae	CA
Black-backed jackal	*Canis mesomelas*	Carnivora	Canidae	CA
Siberian tiger	*Panthera tigris altaica*	Carnivora	Felidae	CA
Bengal tiger	*Panthera tigris tigris*	Carnivora	Felidae	CA
Lion	*Panthera leo*	Carnivora	Felidae	CA
Black leopard	*Panthera pardus*	Carnivora	Felidae	CA
Indochinese leopard	*Panthera pardus delacouri*	Carnivora	Felidae	CA
Jaguar	*Panthera onca*	Carnivora	Felidae	CA
Cheetah	*Acinonyx jubatus*	Carnivora	Felidae	CA
Caracal	*Caracal caracal*	Carnivora	Felidae	CA
Striped hyena	*Hyaena hyaena*	Carnivora	Hyaenidae	CA
Spotted hyena	*Crocuta crocuta*	Carnivora	Hyaenidae	CA
Spotted seal	*Phoca largha*	Carnivora	Phocidae	CA
Sun bear	*Helarctos malayanus*	Carnivora	Ursidae	OC
Brown bear	*Ursus arctos*	Carnivora	Ursidae	OC
Asian black bear	*Ursus thibetanus*	Carnivora	Ursidae	OC
Kinkajou	*Potos flavus*	Carnivora	Procyonidae	OC
Raccoon	*Procyon lotor*	Carnivora	Procyonidae	OC
Hog badger	*Arctonyx collaris*	Carnivora	Mustelidae	OC
Père David's deer	*Elaphurus davidianus*	Artiodactyla	Cervidae	HE
Hairy-fronted muntjac	*Muntiacus crinifrons*	Artiodactyla	Cervidae	HE
Red deer	*Cervus elaphus*	Artiodactyla	Cervidae	HE
Sika deer	*Cervus nippon*	Artiodactyla	Cervidae	HE
Argali	*Ovis ammon*	Artiodactyla	Bovidae	HE
Goitered gazelle	*Gazella subgutturosa*	Artiodactyla	Bovidae	HE
Takin	*Budorcas taxicolor*	Artiodactyla	Bovidae	HE
Onager	*Equus hemionus*	Perissodactyla	Equidae	HE
Hoolock gibbon	*Hoolock spp.*	Primates	Hylobatidae	HE
White-cheeked gibbon	*Nomascus leucogenys*	Primates	Hylobatidae	HE
Black snub-nosed monkey	*Rhinopithecus bieti*	Primates	Cercopithecidae	HE
François's Langur	*Trachypithecus francoisi*	Primates	Cercopithecidae	HE

Table S3 Information on the compositions and relative abundances of gut phages within each group at the order level.

Order	CA	HE	OC	GPCD	GPYA	GPQIN	GPQIO	GPXXL	RP
Caudovirales	99.66%	74.22%	99.38%	99.45%	96.82%	99.09%	91.32%	97.36%	92.24%
Viruses_norank	0.34%	25.72%	0.61%	0.55%	3.18%	0.91%	8.58%	2.64%	7.76%
Kalamavirales	0.00%	0.00%	0.00%	0.00%	0.00%	0.00%	0.09%	0.00%	0.00%
Tubulavirales	0.00%	0.06%	0.01%	0.00%	0.00%	0.00%	0.00%	0.01%	0.00%

Table S4 Information on the compositions and relative abundances of dominant gut phages within each group at the species level.

Species	CA	HE	OC	GPCD	GPYA	GPQIN	GPQIO	GPXXL	RP
Escherichia phage IMM-001	0.00%	0.00%	0.00%	0.00%	0.00%	54.89%	3.54%	0.06%	0.73%
crAssphage cr110_1	43.58%	0.26%	0.00%	0.00%	0.00%	0.00%	0.00%	0.00%	0.00%
crAssphage cr11_1	36.66%	1.39%	0.00%	0.00%	0.00%	0.00%	0.00%	0.00%	0.00%
Pseudomonas phage 201phi2-1	0.00%	0.00%	0.00%	0.00%	0.00%	0.00%	0.01%	59.35%	0.14%
Enterococcus phage EF1	0.79%	0.00%	29.10%	0.00%	0.00%	0.00%	0.00%	0.00%	0.00%
Shigella phage JK32	0.14%	0.01%	0.11%	28.79%	2.94%	0.35%	0.03%	0.41%	0.13%
Bacteriophage sp.	0.30%	25.25%	0.27%	0.20%	1.47%	0.91%	7.63%	2.49%	7.21%
Escherichia phage vB_EcoS_MM01	0.00%	0.00%	0.01%	0.00%	0.00%	5.77%	1.00%	0.01%	0.31%
Escherichia phage vB_EcoS_FP	0.00%	0.00%	0.08%	0.00%	0.00%	5.75%	0.52%	0.01%	0.09%
Salmonella phage vB_Sen_I1	0.00%	0.00%	0.76%	0.00%	3.98%	0.00%	0.00%	0.00%	0.00%
Escherichia phage PD38	0.01%	0.00%	0.26%	0.03%	3.94%	0.00%	0.20%	0.00%	0.00%
Salmonella phage Munch	0.01%	0.00%	0.00%	0.00%	0.00%	2.25%	14.95%	0.06%	0.16%
Escherichia phage CJ19	0.00%	0.00%	0.00%	0.00%	0.00%	4.79%	0.00%	0.00%	0.00%
Escherichia phage St11Ph5	0.02%	0.00%	0.30%	0.02%	3.78%	0.00%	0.18%	0.00%	0.00%
Salmonella phage 8sent1748	0.02%	0.00%	0.65%	0.00%	3.31%	0.00%	0.00%	0.00%	0.00%
Escherichia phage vB_EcoA_4HA11	0.00%	0.00%	0.43%	0.01%	3.01%	0.00%	0.00%	0.00%	0.00%
Aeromonas phage SD04	0.00%	0.00%	0.03%	0.05%	0.90%	0.00%	13.33%	0.00%	0.00%
Salmonella phage SP1a	0.00%	0.00%	0.34%	0.00%	2.40%	0.00%	0.00%	0.00%	0.00%
Enterobacter phage EspM4VN	0.00%	0.00%	0.22%	0.00%	2.42%	0.00%	0.00%	0.00%	0.00%
Enterobacter phage vB_EhoM-IME523	0.00%	0.00%	0.00%	0.00%	0.00%	2.90%	0.01%	0.02%	0.00%
Streptococcus phage P738	0.00%	0.00%	0.87%	0.01%	1.99%	0.00%	0.00%	0.00%	0.00%
Salmonella phage vB_SalS_ABTNLsp4	0.00%	0.00%	0.36%	0.00%	2.07%	0.00%	0.00%	0.00%	0.00%
Escherichia phage vB_EcoD_SU57	0.00%	0.00%	0.14%	0.00%	0.00%	2.51%	0.33%	0.01%	0.17%
Escherichia phage vB_EcoP_SP5M	0.03%	0.00%	0.09%	0.01%	2.00%	0.00%	0.60%	0.01%	0.03%
Escherichia phage ESCO13	0.14%	0.00%	1.92%	0.05%	1.19%	0.04%	0.72%	0.00%	0.00%
Salmonella phage Stp1	0.00%	0.16%	0.28%	0.00%	1.84%	0.00%	0.03%	0.00%	0.00%
Escherichia phage Greed	0.00%	0.00%	0.02%	0.06%	0.85%	0.00%	7.27%	0.00%	0.00%
Klebsiella phage vB_KpnM_IME346	0.88%	0.00%	0.27%	0.00%	1.15%	0.00%	0.00%	0.01%	0.00%
Escherichia phage vB_EcoM_G2248	0.00%	0.00%	0.04%	7.45%	0.76%	0.07%	0.00%	0.10%	0.00%
Pseudomonas phage Phabio	0.00%	0.00%	0.00%	0.01%	0.00%	0.00%	0.00%	4.14%	8.70%
Enterococcus phage Q69	0.00%	0.00%	0.00%	0.00%	0.00%	0.00%	0.00%	0.01%	19.44%

Figure S1 Hierarchical clustering analysis based on the composition of gut bacteria and phage communities among all groups. CA, carnivorous mammal. GPCD, captivity giant panda in Chengdu. GPQIN, wild giant panda in Qinling. GPQIO, wild giant panda in Qionglai. GPXXL, wild giant panda in Xiaoxiangling. GPYA, captivity giant panda in Ya'an. HE, herbivorous mammal. OC, omnivorous mammal. RP, wild red panda in Xiaoxiangling.

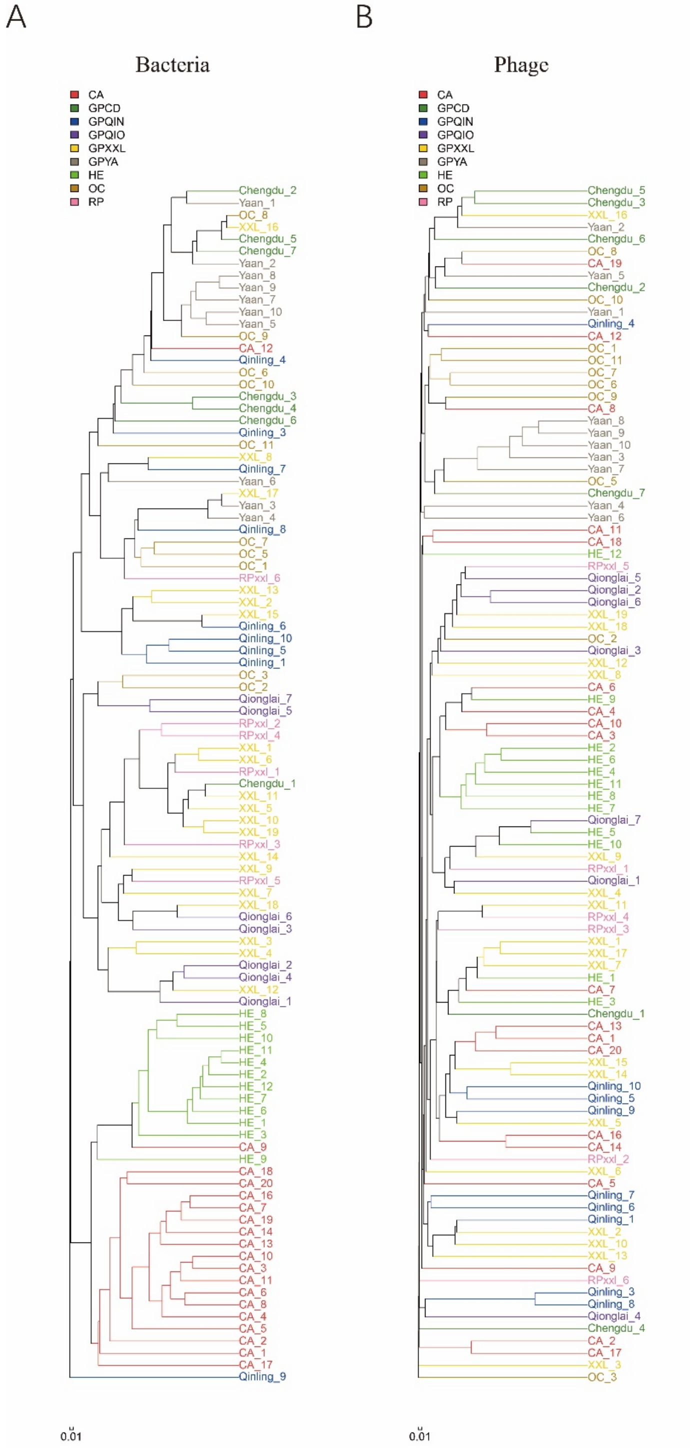

Figure S2 Co-occurrence network analysis of bacteria (A), and phage (B) communities in all groups. The nodes representing taxonomic groups of bacterial and phage are color-coded based on phylum and family, respectively. The size of the node represents the number of connections (degree value). Different colored edges to represent positive (green) and negative (red) correlations between nodes. GPCD, captivity giant panda in Chengdu. GPYA, captivity giant panda in Ya'an. GPQIN, wild giant panda in Qinling. GPQIO, wild giant panda in Qionglai. GPXXL, wild giant panda in Xiaoxiangling. RP, wild red panda in Xiaoxiangling. CA, carnivorous mammal. OC, omnivorous mammal. HE, herbivorous mammal.

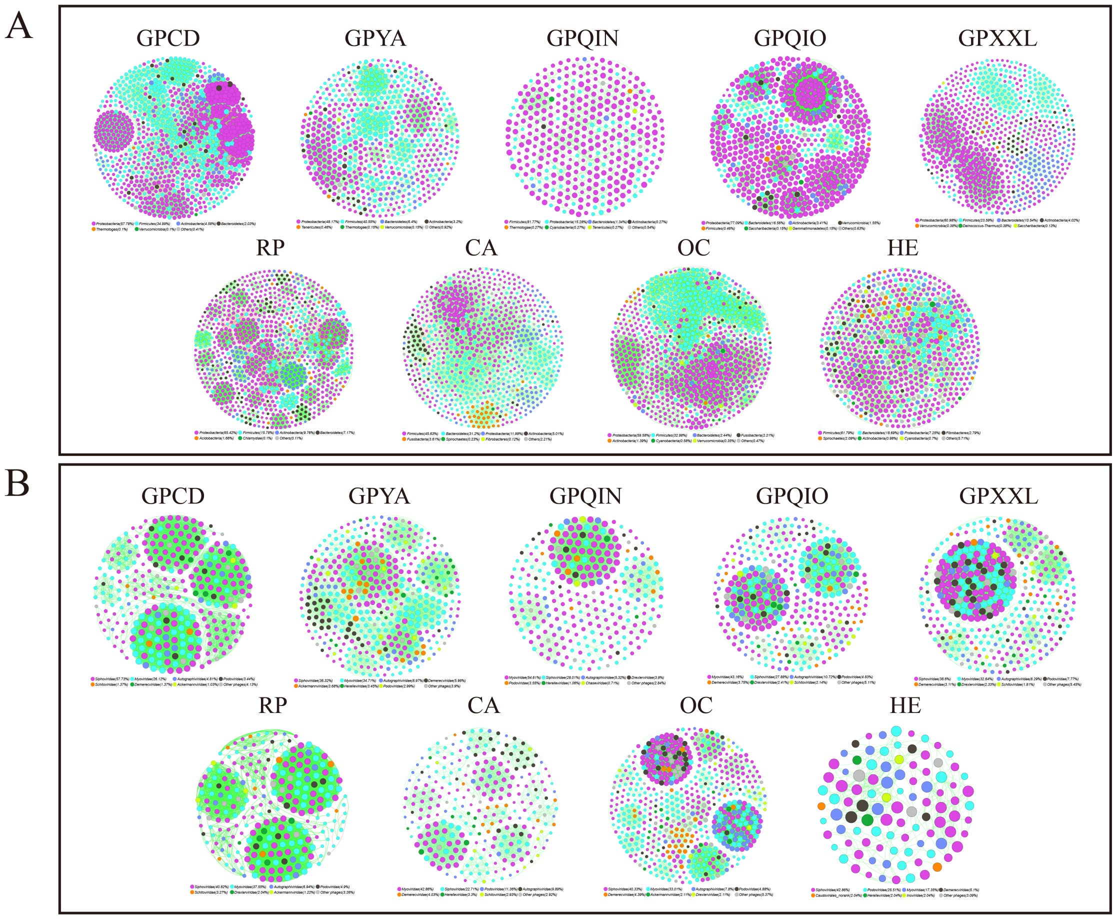

Figure S3 Modular structure of co-occurring networks of bacterial (A), phage (B), and bacterial-phage communities in all groups. The nodes affiliated with different modules were assigned distinct colors. The size of the node represents the number of connections (degree value). Different colored edges to represent positive (green) and negative (red) correlations between nodes. GPCD, captivity giant panda in Chengdu. GPYA, captivity giant panda in Ya'an. GPQIN, wild giant panda in Qinling. GPQIO, wild giant panda in Qionglai. GPXXL, wild giant panda in Xiaoxiangling. RP, wild red panda in Xiaoxiangling. CA, carnivorous mammal. OC, omnivorous mammal. HE, herbivorous mammal.

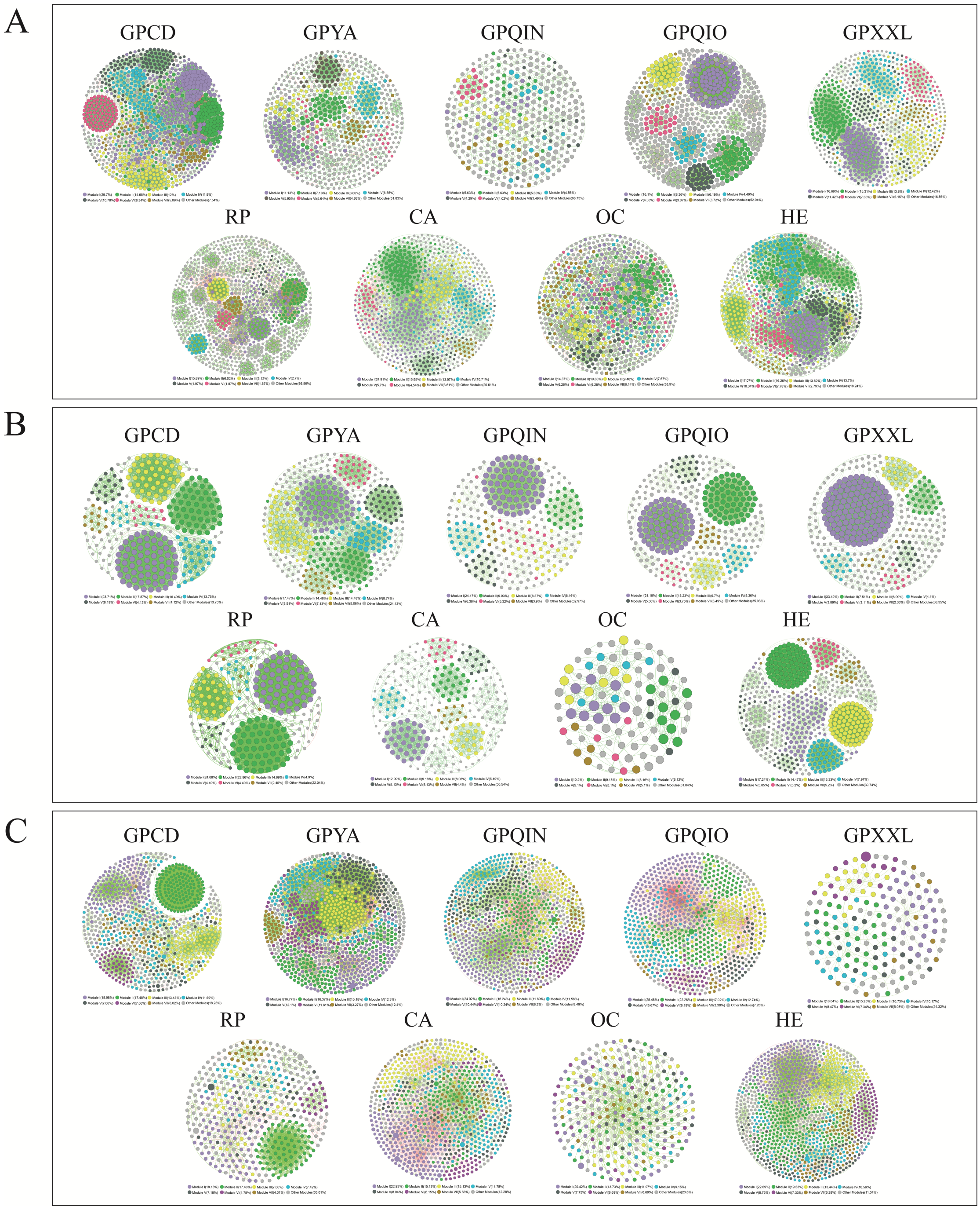

Figure S4 Cohesion levels of gut bacteria, phage, and bacteria-phage communities was assessed across four different dietary preferences animal groups (carnivores, herbivores, omnivores, and bamboo-eating animals). The positive cohesion in (A) bacteria, (D) phage, and (G)bacteria-phage networks. The negative cohesion in (B) bacteria, (E) phage, and (H)bacteria-phage networks. The total cohesion (the sum of the absolute values of the positive and negative cohesion values) in (C) bacteria, (F) phage, and (I)bacteria-phage networks. The significance levels were calculated by Kruskal-Wallis test. GPCD, captivity giant panda in Chengdu. GPYA, captivity giant panda in Ya'an. GPQIN, wild giant panda in Qinling. GPQIO, wild giant panda in Qionglai. GPXXL, wild giant panda in Xiaoxiangling. RP, wild red panda in Xiaoxiangling. CA, carnivorous mammal. OC, omnivorous mammal. HE, herbivorous mammal.

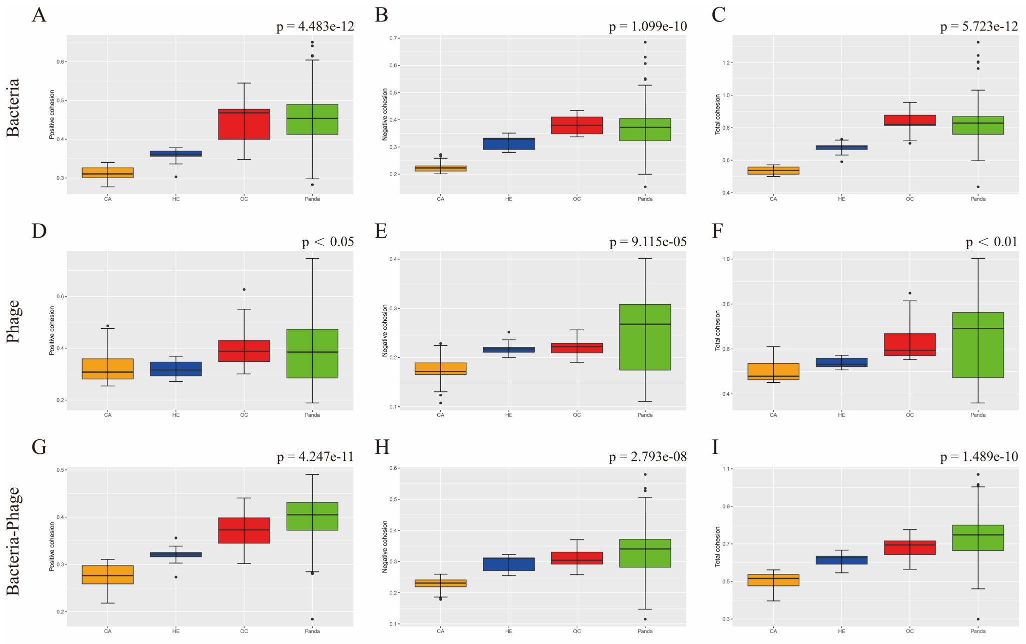

Reference

Guo, W., Mishra, S., Wang, C., Zhang, H., Ning, R., Kong, F., Zeng, B., Zhao, J., and Li, Y. (2019). Comparative Study of Gut Microbiota in Wild and Captive Giant Pandas (Ailuropoda melanoleuca). Genes *10*.

Wu, Q., Wang, X., Ding, Y., Hu, Y., Nie, Y., Wei, W., Ma, S., Yan, L., Zhu, L., and Wei, F. (2017). Seasonal variation in nutrient utilization shapes gut microbiome structure and function in wild giant pandas. Proceedings of the Royal Society B: Biological Sciences *284*, 20170955.

Zhang, W., Liu, W., Hou, R., Zhang, L., Schmitz-Esser, S., Sun, H., Xie, J., Zhang, Y., Wang, C., Li, L*., et al.* (2018). Age-associated microbiome shows the giant panda lives on hemicelluloses, not on cellulose. The ISME journal *12*, 1319-1328.

Zhu, L., Wu, Q., Deng, C., Zhang, M., Zhang, C., Chen, H., Lu, G., and Wei, F. (2018a). Adaptive evolution to a high purine and fat diet of carnivorans revealed by gut microbiomes and host genomes. Environmental Microbiology *20*, 1711-1722.

Zhu, L., Yang, Z., Yao, R., Xu, L., Chen, H., Gu, X., Wu, T., and Yang, X. (2018b). Potential Mechanism of Detoxification of Cyanide Compounds by Gut Microbiomes of Bamboo-Eating Pandas. mSphere *3*, 10.1128/msphere.00229-00218.
